# Metabolic Footprint of Diabetes: A Multiplatform Metabolomics Study in an Epidemiological Setting

**DOI:** 10.1371/journal.pone.0013953

**Published:** 2010-11-11

**Authors:** Karsten Suhre, Christa Meisinger, Angela Döring, Elisabeth Altmaier, Petra Belcredi, Christian Gieger, David Chang, Michael V. Milburn, Walter E. Gall, Klaus M. Weinberger, Hans-Werner Mewes, Martin Hrabé de Angelis, H.-Erich Wichmann, Florian Kronenberg, Jerzy Adamski, Thomas Illig

**Affiliations:** 1 Institute of Bioinformatics and Systems Biology, Helmholtz Zentrum München, German Research Center for Environmental Health, Neuherberg, Germany; 2 Faculty of Biology, Ludwig-Maximilians-Universität, Planegg-Martinsried, Germany; 3 Institute of Epidemiology, Helmholtz Zentrum München, German Research Center for Environmental Health, Neuherberg, Germany; 4 Chenomx, Edmonton, Alberta, Canada; 5 Metabolon Inc., Durham, North Carolina, United States of America; 6 Biocrates Life Sciences AG, Innsbruck, Austria; 7 Department of Genome-Oriented Bioinformatics, Life and Food Science Center Weihenstephan, Technische Universität München, Freising-Weihenstephan, Germany; 8 Institute of Experimental Genetics, Genome Analysis Center, Helmholtz Zentrum München, German Research Center for Environmental Health, Neuherberg, Germany; 9 Institute of Experimental Genetics, Life and Food Science Center Weihenstephan, Technische Universität München, Freising-Weihenstephan, Germany; 10 Institute of Medical Informatics, Biometry and Epidemiology, Ludwig-Maximilians-Universität and Klinikum Grosshadern, Munich, Germany; 11 Division of Genetic Epidemiology, Department of Medical Genetics, Molecular and Clinical Pharmacology, Innsbruck Medical University, Innsbruck, Austria; INSERM, France

## Abstract

**Background:**

Metabolomics is the rapidly evolving field of the comprehensive measurement of ideally all endogenous metabolites in a biological fluid. However, no single analytic technique covers the entire spectrum of the human metabolome. Here we present results from a multiplatform study, in which we investigate what kind of results can presently be obtained in the field of diabetes research when combining metabolomics data collected on a complementary set of analytical platforms in the framework of an epidemiological study.

**Methodology/Principal Findings:**

40 individuals with self-reported diabetes and 60 controls (male, over 54 years) were randomly selected from the participants of the population-based KORA (Cooperative Health Research in the Region of Augsburg) study, representing an extensively phenotyped sample of the general German population. Concentrations of over 420 unique small molecules were determined in overnight-fasting blood using three different techniques, covering nuclear magnetic resonance and tandem mass spectrometry. Known biomarkers of diabetes could be replicated by this multiple metabolomic platform approach, including sugar metabolites (1,5-anhydroglucoitol), ketone bodies (3-hydroxybutyrate), and branched chain amino acids. In some cases, diabetes-related medication can be detected (pioglitazone, salicylic acid).

**Conclusions/Significance:**

Our study depicts the promising potential of metabolomics in diabetes research by identification of a series of known and also novel, deregulated metabolites that associate with diabetes. Key observations include perturbations of metabolic pathways linked to kidney dysfunction (3-indoxyl sulfate), lipid metabolism (glycerophospholipids, free fatty acids), and interaction with the gut microflora (bile acids). Our study suggests that metabolic markers hold the potential to detect diabetes-related complications already under sub-clinical conditions in the general population.

## Introduction

Type 2 diabetes mellitus is a complex disease [1], which is characterized by abnormal hepatic glucose output, insulin resistance and impaired insulin production [2], [3]. It may be assumed that in individuals with type 2 diabetes many metabolic pathways are likely to be affected and presumably play a role in their overall metabolic dysfunction. Thus, the identification of new biomarkers and pathways can improve the characterization of pathophysiological alterations associated with the disease condition [4]. Metabolomics is the rapidly evolving field of the comprehensive measurement of ideally all endogenous metabolites in a biological fluid [5], [6], [7], [8], [9], [10]. Changes in metabolic profiles are a potential source of such biomarkers [11], [12], [13], [14]. We have previously reported an analysis of targeted quantitative metabolomics, where we have shown that many known and novel observations of metabolic changes may be discovered using such a metabolomics approach and that targeted quantitative metabolomics provides a functional readout of the metabolic state of diabetic mice under medication [15]. The method has the power to identify perturbations of the body's metabolic homeostasis and thereby offers access to markers of metabolic pathways that are impacted by the disease and/or medication. Such markers could help physicians to identify patients at high risk for specific complications, thereby allowing a personalized approach to monitoring and preventing progression to costly co-morbidities.

The principal concept of metabolomics being able to find some metabolites differing in a control and a type 2 diabetic group is established. It is not our goal here to show this once again. The questions we ask are rather “How well are different approaches suited to attain this goal?” and “What are optimal settings under which such studies can be successful?”. Others have already investigated these questions before [16], [17], [18]. However, we believe that this topic is much too complex than to be answered fully in a single study. For instance, the work described in the recent paper in this journal by Lanza et al. [19] covers only a small patient group of 7 cases and 7 controls. Our study, in contrast is based on 40 cases and 60 controls from an epidemiological cohort. Work reviewed recently by Madsen et al. [20] overlaps to some extent with our study, but none of them address aspects related to sub-clinical signals in a general population. Our focus is on participants from epidemiological studies rather than on patients under clinical conditions. Herein, we identify a series of differentially “expressed” metabolites that associate with diabetes under sub-clinical conditions in the general population. This question has not been addressed to this extent by any published paper. In particular, we see our work as a pilot that bears the potential of being scaled up to much larger sample sizes, since population studies such as KORA eventually provide access to much larger sample sizes, taken under rigorous standardized blood sample collection conditions in dedicated study centers (e.g. overnight fasting, standard protocol for serum and plasma preparation, storage in liquid nitrogen until measurement). These kinds of samples generally have not been available from clinical studies until recently. It is in this light that we provide here a proof of concept that metabolomics can uncover key metabolites differing in a control and a type 2 diabetic group.

Obtaining the most comprehensive coverage of the metabolome experimentally is key to identifying novel markers of diabetes using the metabolomics approach [4], [21], [22]. Over the past two years we have conducted experiments with three different fee-for-service providers of quantitative metabolomics: *Biocrates Life Sciences AG* (Austria), *Chenomx Inc.* (Canada), and *Metabolon Inc.* (USA) by submitting to each of them blood samples from the same 100 participants of the population-based study KORA (Cooperative Health Research in the Region of Augsburg) for metabolic characterization. The methods and the metabolic profiling platforms of the three companies are mostly complementary, but also provide some overlap which allows for a certain level of cross-validation. Biocrates and Metabolon apply tandem mass spectrometry (MS), whereas Chenomx specializes in nuclear magnetic resonance (NMR) spectroscopy. Furthermore, Metabolon takes a non-targeted approach, combining gas (GC) and liquid phase (LC) chromatography; whereas, Biocrates applies targeted metabolomics, based on pre-selected MRM pairs and isotope labeled internal standards, with many of their methods being based on rapid direct flow injection (FIA), however with capacity to carry out multiple GC-MS and LC-MS protocols. NMR has the advantage of leaving the sample intact, but requires much larger (10-100x) sample volumes. FIA-MS is especially adapted to high-throughput assays when large populations or time-series with many data points are investigated. One example is newborn screening for inborn errors of metabolism [23] where the power of metabolomics has been shown for the early detection of many monogenic disorders [24]. A combination of LC-MS and GC-MS assures a maximum coverage of a wide metabolite spectrum, but is more likely adapted to applications for metabolite biomarker discovery studies [25]. The metabolic approaches used here are thus technologically distinct but overlapping in targets identified.

Here we present the results from these studies, which implement a case/control design in males aged over 55 years with type 2 diabetes. Because no single analytic technique covers the entire spectrum of the human metabolome, we ask what kind of results can presently be obtained in the field of diabetes research when combining metabolomics data collected on a complementary set of platforms in the context of the general human population. By utilizing this comprehensive biochemical profiling approach, we seek to identify metabolites with different concentrations in patients with diabetes and in healthy controls, and thereby allowing new insights into the pathophysiological progression of this important metabolic disease under subclinical conditions.

## Materials and Methods

### Ethics statement

Written informed consent has been given by all participants. This study has been approved by the ethics committee of the Bavarian Medical Association (Bayerische Landesärztekammer).

### Study population

The participants of this study were selected from the KORA F3 cohort study [26], which is an extensively phenotyped and genome-wide genotyped sample from the general population, conducted in 2004/2005. The total KORA F3 study (n = 3006) comprises male and female individuals aged between 35 and 84 years, who are residents of the city and region of Augsburg, Southern Germany. Standardized examinations and tests that were applied to the study participants have been described in detail elsewhere [27], including clinical biochemistry and extensive coverage of different life-style parameters by questionnaires. To reduce the degree of natural variation in the data-set, we limited this study to the “male above 54 years” sub-population. 40 individuals with self-reported “type 2 diabetes”, validated by a physician diagnosis, and 60 randomly age matched healthy individuals who were fasting at the time of blood collection were selected from that sub-population as case/control groups for this study. A comparison of the basic characteristics of the diabetes and the control groups are presented in Table 1.

**Table 1:** Characteristics** of the diabetic and the control group.

	Diabetes	Non-diabetes	p-value
N	40	60	
Age range	55–79	55–79	
Age (years)	67.7 [7.2]	65.6 [6.4]	>0.05
Cholesterol (mg/dl)	197.17 [40.6]	219.17 [35.4]	0.0066
HDL (mg/dl)	50.50 [16.0]	56.71 [12.9]	0.041
LDL (mg/dl)	120.50 [37.5]	137.81 [35.4]	0.026
Triglycerides (mg/dl)	208.39 [258.3]	157.42 [106.4]	>0.05
HbA1c (%)*	5.95 [0.72]	5.29 [0.37]	3.0×10^−8^
BMI*	30.01 [3.6]	28.31 [3.4]	0.019
Waist-Hip-Ratio*	0.990 [0.049]	0.957 [0.054]	0.0021

*determined at baseline 2004/2005, about 1–2 years prior to sampling for metabolomics.

**mean [standard deviation].

### Sample collection

For collection of blood samples for metabolic analysis, F3 study participants were invited again in 2006. To avoid variation due to circadian rhythm, blood was drawn in the morning between 8 and 10 am after a period of overnight fasting. After venous puncture, material was immediately horizontally shaken (10 min), and for the serum tubes, followed by a 40 min resting period at room temperature to obtain complete coagulation. After centrifugation (2000 g; 4°C) serum was aliquoted and kept for 2–4 hours on ice, after which it was flash-frozen to −80°C until analysis. EDTA-blood was horizontally shaken (15 min) and thereafter centrifuged at 4°C for 10 min at 2000 g. Plasma was transferred to new tubes without aspirating blood cells. Aliquots were kept for 2–4 hours at 4°C and then stored at −80°C.

### Metabolomics measurements

Metabolite detection and quantification was conducted by the metabolomics providers *Biocrates Life Sciences AG* (Innsbruck, Austria), *Chenomx Inc.* (Edmonton, Canada), and *Metabolon Inc.* (Durham, USA). The companies had no access to phenotype information that would have permitted any data pre-filtering other than objective quality control for measurement errors based on internal controls and duplicates. All metabolomics data was used as received, no data correction was applied, and no data points were removed. The quality control process applied by these companies is described in Text S1.

#### Biocrates platform

A targeted profiling scheme was used to quantitatively screen for known small molecule metabolites using multiple reaction monitoring, neutral loss and precursor ion scans. For the present study, a subset of the available analytical methods was selected: 201 metabolites covering the compound classes amino acids, biogenic amines and polyamines, reducing mono- and oliogosaccharides, glycerophospho- and sphingolipids, eicosanoids and other oxidized polyunsaturated fatty acids were detected and quantified. Absolute quantitation of the metabolites in the biological sample was achieved by reference to appropriate internal standards which are structurally identical but labeled with stable isotopes such as deuterium, ^13^C, or ^15^N. The method has been proven to be in conformance with FDA-Guideline "Guidance for Industry - Bioanalytical Method Validation (May 2001”), which implies proof of reproducibility within a given error range. Concentrations of all analyzed metabolites are reported in µM (except for eicosanoid concentrations which are reported in nM units in the supplementary data files). This data set is a subset of another data set that has already been analyzed in the context of a genome wide association study [28], for the discovery of biomarkers of smoking [29] and a metabolome wide association with coffee consumption [30]. The full dataset comprises additional data from healthy individuals who have not been analyzed on the two other metabolomics platforms used here, and data for metabolites with a high fraction of missing values (>10%) was excluded here to avoid spurious associations due to small sample sizes.

#### Chenomx platform

Samples were prepared for NMR analysis using Chenomx SOP (CMX002). This platform included micro-filtration of samples using a 3 kDa MWCO filter and the addition of (3-trimethylsilyl) propanesulfonic acid (DSS) as an internal standard. Spectra were acquired on a 600 MHz Varian INOVA spectrometer. Thirty-two transients were recorded at a temperature of 298 K. Spectra were processed and CNX files were generated using the Processor module in Chenomx NMR Suite 5.11. Metabolites were identified and quantified using the Profiler and Library Manager modules in Chenomx NMRSuite 5.11 (library version: pH 6–8, containing 292 metabolites). The methods for identification and quantification of the metabolites were determined as previously described [31]. Altogether, 24 metabolites were identified in the EDTA plasma samples, including alcohols, amides, amines, amino acid derivatives, amino acids, aromatic compounds, fatty acids, food/drug components, organic acids and sugars. For this study, due to the fact that only 500 µl of prefiltered plasma were available, samples had to be diluted by a minimum of 2 times its original volume, thereby reducing the overall sensitivity of the method and the number of detected metabolites.

#### Metabolon platform

Metabolon developed a platform that integrates the chemical analysis, including identification and relative quantification, data reduction, and quality assurance components of the process. The analytical platform incorporates two separate ultra-high performance liquid chromatography/tandem mass spectrometry (UHPLC/MS/MS2) injections and one GC/MS injection per sample. The UHPLC injections are optimized for basic species and acidic species. This approach permitted the detection of 257 small molecules, with total instrument analysis time of 24 min (two injections at 12 min each), while maintaining a median process variability for all compounds of 9%. Metabolon also has the ability to measure additional compounds that do not currently have a chemical standard, but this was not done in this study. The resulting MS/MS^2^ data were searched against an in-house generated authentic standard library that included retention time, molecular weight (m/z), preferred adducts, and in-source fragments, includingtheir associated MS/MS spectra for all molecules in the library. The library allowed for the rapid and high-confidence identification of the experimentally detected molecules based on a multi-parameter match basis without need for additional analyses. This integrated platform enabled the high-throughput collection and relative quantitative analysis of analytical data and identified a large number and broad spectrum of molecules with a high degree of confidence [25]. The Metabolon platform has, among other studies, been successfully applied in the analysis of the adult human plasma metabolome [32] identification of sarcosine as a biomarker for prostate cancer [33], and biomarkers of insulin resistance in a nondiabetic population [34].

### Statistical analysis

The statistical analysis system R (Version 2.6.0, http://www.r-project.org/) was used for metabolome-wide analyses and SPSS for Windows (Version PASW 17.0, Chicago: SPSS Inc.) was used on a case-by-case basis. Statistical association queries with phenotype “diabetes” were tested in a linear model using body mass index (BMI) as a covariate. Partial eta-square values are reported as a measure of effect size. To correct for multiple testing, the positive false discovery rate was used by computing q-values after Storey and Tibshirani [35]. Motivated by our previous observations [15], [28] that the use of ratios may lead to a strong reduction in the overall variance, we computed and tested all possible pairs of metabolite concentration ratios. A strong reduction in p-value (p-gain) indicates that two metabolites may be linked by a metabolic pathway that is impacted by the diabetes state of the patients. Dependant variables (metabolite concentrations and concentration ratios) were log-scaled prior to computing the statistics. Note that testing ratios between two metabolites *a* and *b* is independent of their order, as log(*a*/*b*) = −log(*b*/*a*), which halves multiple testing burden. For concentration ratios, only statistically significant associations that display a p-gain greater than 241 are further analyzed. This limit is considered as a Bonferroni-type conservative cut-off for identifying those metabolite concentration pairs for which the use of ratios strongly improves the strength of association. Un-scaled variables and a parameter-free Kendall test were also used to confirm the statistical robustness of the associations. P-values for these tests are reported as supplementary data.

### Covariates

In order to identify a metabolic signature associated with anti-diabetic treatment at a statistically significant level, the sample size of our study is still quite small. However, in order to test for potentially confounding factors, we analyzed the impact of the following diabetes-related treatments on our results: lipid-lowering medication (excluding plant based), blood pressure lowering medication, antidiabetic medication (insulin or oral), statin treatment, insulin therapy, oral antidiabetic medication, calcium antagonists, beta blockers, diuretics, and ACE inhibitors. We also tested the influence of other factors, including age, smoking habits, body mass index (BMI), waist-hip-ratio, alcohol consumption, physical activity, and myocardial infarction. Finally, we tested the effect of antidiabetic medication (insulin or oral), insulin therapy, and oral antidiabetic medication on the metabolite spectrum for the diabetic group alone. The details of these additional statistical tests are provided as supplemental data.

## Results

### The metabolomic dataset

**Figure 1:**
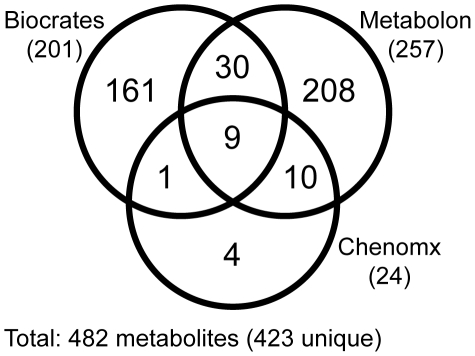
Venn diagram showing the number of metabolites common to all three platforms, to two platforms and metabolites detected specifically by one platform. The identity of the individual metabolites that are measured on each platform is provided in Table S2. Note that the metabolites metabolites that are quantified uniquely on the Biocrates platform carry specific information on the lipid side-chain composition of the different phospholipid classes (sometimes also referred to as lipidomics). The Metabolon platform, in contrast, provides a wider non-targeted, but semi-quantitative coverage of the general metabolome. NMR presently allows quantifying only a smaller set of metabolites, but this at a much higher degree of reproducibility, faster, and without specific sample preparation.

In the final data set, 482 distinct values of absolute (Biocrates and Chenomx) or relative (Metabolon) metabolite concentrations were available for analysis (Figure 1). 50 metabolites were quantified on more than one platform: Ten metabolites were measured on the Biocrates and the Chenomx platform, 39 on the Biocrates and the Metabolon platform, 19 on the Chenomx and the Metabolon platform, including 9 metabolites that were measured on all three platforms. Thus, a total of 423 unique metabolites were quantified on at least one platform. Comparisons of the 68 duplicate measurements revealed that correlation coefficients (R) between the platforms showed a median correlation coefficient of 0.61 (Figure S1). In three cases no correlation was found, indicating that the different techniques may be measuring different metabolites here de facto. In other cases, very strong correlation (up to R = 0.95) between at least two of the platforms was observed, showing that in principle cross-platform replication may be possible (Figure S2; data provided as Table S1).

### Associations of metabolites with diabetes

Table 2 provides metabolites that display significant differences between the case and the control group after controlling for multiple testing using the positive false discovery rate [35] (q-value <0.05). In total, associations with 482 metabolites were tested (423 unique), with 201 metabolites from Biocrates, 257 from Metabolon, and 24 from Chenomx. 32 associations display a positive false discovery rate that is smaller than 5%, and 114 associations are significant with a p-value smaller than 0.05 (data provided in Table S2). Table 3 lists metabolite pairs that display significant increase in the strength of association when using ratios (p-gain>241), thereby indicating that these metabolite pairs are possibly linked through some diabetes-related metabolic or regulatory process. The full set of associations is provided in Table S3.

**Table 2:** List of selected metabolites that associate with diabetes at q-values <0.05 using log-scaled metabolite concentrations and assuming a linear model; %change is the increase or decrease of the mean in the diabetes group with respect to the control group; eta^2^ is the proportion of the total variance that can be explained by the factor “diabetes” in the linear model; N is the number of valid data points that entered the analysis; the platform on which the corresponding metabolite was measured is indicated by the first letter of the provider: B = Biocrates, C = Chenomx, M = Metabolon.

Metabolite	Pathway	%-change	N	p-value	q-value	eta^2^
1,5-anhydroglucitol [M]	Sugar	−37.8%	98	5.1×10^−6^	3.2×10^−4^	19.6%
desoxyhexose (DH) [B]	Sugar	40.2%	99	1.3×10^−6^	9.4×10^−5^	21.6%
glucose [C]	Sugar	39.3%	100	5.0×10^−8^	7.4×10^−6^	26.3%
glucose [M]	Sugar	28.8%	99	2.5×10^−9^	1.1×10^−6^	30.8%
H3-HNAc2-NANA [B]	Sugar	90.0%	99	2.4×10^−8^	5.3×10^−6^	27.6%
HNAC [B]	Sugar	18.0%	99	6.2×10^−5^	2.8×10^−3^	15.3%
HNAc-H2-dH [B]	Sugar	64.8%	99	8.2×10^−8^	9.1×10^−6^	25.8%
uronic acid [B]	Sugar	45.8%	99	8.0×10^−4^	1.7×10^−2^	11.0%
dihexose (H2) [B]	Sugar	65.2%	99	2.7×10^−5^	1.5×10^−3^	16.7%
mannose [M]	Sugar	34.9%	99	2.4×10^−7^	2.1×10^−5^	24.2%
caproate (6:0) [M]	Fatty acid, saturated, even	−16.1%	99	1.5×10^−3^	2.7×10^−2^	9.9%
heptanoate (7:0) [M]	Fatty acid, saturated, odd	−15.4%	99	5.2×10^−4^	1.3×10^−2^	11.7%
pelargonate (9:0) [M]	Fatty acid, saturated, odd	−12.6%	99	1.9×10^−3^	2.9×10^−2^	9.5%
glycerophosphorylcholine [M]	Glycerolipid	−16.5%	98	4.2×10^−4^	1.2×10^−2^	12.2%
PC a C20:4 (alt) [B]	Glycerolipid	−19.1%	100	1.4×10^−3^	2.7×10^−2^	9.9%
PC aa (OH, COOH) C28:4 [B]	Glycerolipid	−16.4%	100	1.7×10^−3^	2.8×10^−2^	9.6%
PC aa C34:4 [B]	Glycerolipid	−26.0%	100	6.5×10^−4^	1.5×10^−2^	11.2%
SM C14:0 [B]	Sphingolipid	−18.7%	100	1.2×10^−3^	2.3×10^−2^	10.2%
SM C22:2 [B]	Sphingolipid	−16.3%	100	3.3×10^−3^	4.6×10^−2^	8.5%
creatinine [M]	Creatine	19.4%	99	3.2×10^−4^	9.6×10^−3^	12.5%
glutamylvaline [M]	Dipeptide	26.4%	99	2.9×10^−4^	9.6×10^−3^	12.7%
gamma-glutamylisoleucine [M]	g-glutamyl	27.8%	92	6.7×10^−4^	1.5×10^−2^	12.1%
3-hydroxybutyrate (BHBA) [M]	Ketone bodies	53.9%	99	1.9×10^−3^	2.9×10^−2^	9.5%
phenylacetylglutamine [M]	Phenylalanine & tyrosine	76.5%	99	6.2×10^−5^	2.8×10^−3^	15.3%
phenylalanine [B]	Phenylalanine & tyrosine	9.0%	100	2.4×10^−3^	3.6×10^−2^	9.0%
3-indoxyl sulfate [M]	Tryptophan	42.8%	99	1.7×10^−4^	6.1×10^−3^	13.7%
kynurinine [B]	Tryptophan	21.8%	100	5.0×10^−4^	1.3×10^−2^	11.7%
homocitrulline [M]	Urea cycle; arginine-, proline-,	73.4%	85	3.1×10^−4^	9.6×10^−3^	14.6%

**Table 3:** List of selected metabolite concentration ratios that associate with diabetes at q-values <0.05 and that display a significant increase in the strength of association (p-gain>241) after Bonferroni correction; based on log-scaled metabolite concentration ratios and assuming a linear model.

Lower in diabetes	Higher in diabetes	Pathway	Pathway	N	p-value	q-value	eta2	p-gain
1,5-anhydroglucitol [M]	desoxyhexose (DH) [B]	Sugar	Sugar	97	4.7×10^−9^	9.0×10^−6^	30.5%	271.6
1,5-anhydroglucitol [M]	Dihexose (H2) [B]	Sugar	Sugar	97	2.0×10^−8^	1.7×10^−5^	28.3%	254.3
PC aa C34:4 [B]	3-indoxyl sulfate [M]	Glycerolipid	Tryptophan	99	2.1×10^−7^	6.4×10^−5^	24.3%	775.1
pro-hydroxy-pro [M]	phenylacetylglutamine [M]	Dipeptide	Phenylalanine & tyrosine	99	2.5×10^−7^	7.1×10^−5^	24.1%	246.4
heptanoate (7:0) [M]	glutamylvaline [M]	Fatty acid, saturated, odd	Dipeptide	99	4.0×10^−7^	9.8×10^−5^	23.4%	736.8
SM C14:0 [B]	3-indoxyl sulfate [M]	Sphingolipid	Tryptophan	99	5.3×10^−7^	1.2×10^−4^	22.9%	310.7
cysteine [M]	glutamylvaline [M]	Cysteine	Dipeptide	95	6.6×10^−7^	1.4×10^−4^	23.4%	442.9
cysteine [M]	3-indoxyl sulfate [M]	Cysteine	Tryptophan	95	6.8×10^−7^	1.4×10^−4^	23.4%	242.7
caproate (6:0) [M]	glutamylvaline [M]	Fatty acid, saturated, even	Dipeptide	99	7.2×10^−7^	1.5×10^−4^	22.5%	410.3
cysteine [M]	creatinine [M]	Cysteine	Creatine	95	1.1×10^−6^	2.1×10^−4^	22.6%	289.5
cysteine [M]	Uronic Acid [B]	Cysteine	Sugar	94	1.4×10^−6^	2.4×10^−4^	22.5%	572.3
cysteine [M]	gamma-glutamylisoleucine [M]	Cysteine	g-glutamyl	88	2.7×10^−6^	3.8×10^−4^	22.7%	249.0
cysteine [M]	erythronate [M]	Cysteine	Aminosugars	93	4.3×10^−6^	5.3×10^−4^	20.8%	901.2
cysteine [M]	erythritol [M]	Cysteine	Sugar, sugar substitute, starch	95	7.5×10^−6^	7.9×10^−4^	19.5%	656.4
cysteine [M]	N-acetylalanine [M]	Cysteine	Valine & (Iso)Leucine	94	1.5×10^−5^	1.3×10^−3^	18.6%	379.9
arachidonate (20:4n6) [M]	Isoleucine [C]	Fatty acid, polyene	Valine & (Iso)Leucine	99	2.0×10^−5^	1.6×10^−3^	17.2%	274.4
uridine [M]	2-methylbutyroylcarnitine [M]	Pyrimidine	Carnitine	87	2.5×10^−5^	1.8×10^−3^	19.0%	245.2
beta-hydroxyisovalerate [M]	PE aa C34:2 [B]	Valine & (Iso)Leucine	Glycerolipid	60	2.6×10^−5^	1.9×10^−3^	26.5%	949.0
beta-hydroxyisovalerate [M]	PE aa C36:2 [B]	Valine & (Iso)Leucine	Glycerolipid	60	4.5×10^−5^	2.8×10^−3^	25.2%	546.0
beta-hydroxyisovalerate [M]	PE aa C38:4 [B]	Valine & (Iso)Leucine	Glycerolipid	60	8.3×10^−5^	4.3×10^−3^	23.6%	294.7
uridine [M]	3-methyl-2-oxovalerate [M]	Pyrimidine	Valine & (Iso)Leucine	99	9.4×10^−5^	4.8×10^−3^	14.6%	403.4

**Figure 2:**
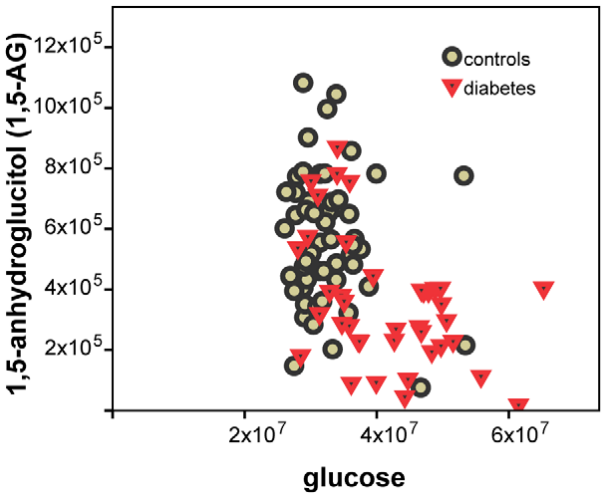
1,5-AG and glucose (measured on Metabolon platform). Lower 1,5-AG concentrations at higher glucose levels in participants with diabetes when compared to the control group display the current role of 1,5-AG as a marker for glycemic control in patients with diabetes.

In line with our expectations, the strongest positive associations with diabetes are observed for the numerous sugar metabolites that were observed on the three platforms. Concentrations of glucose, mannose, desoxyhexose (primarily deoxyglucose), uronic acid (primarily glucuronic acid), dihexose (primarily maltose), and several products from the biosynthesis or the degradation of glycosylated proteins or glycolipids (H3-HNAc2-NANA, HNAC, HNAc-H2-dH) are all found increased by up to 90% in the diabetes group (p = 1.3×10^−4^ to 2.5×10^−9^). Furthermore, there is a significant decrease of average 1,5-anhydroglucitol concentrations by 37.8% (p = 5.1×10^−6^) in participants with diabetes when compared to the control group (Figure 2).

Among the numerous glycerophospholipids that are determined on the Biocrates platform, the phosphatidylcholines PC_aa_C34:4 (p = 6.5×10^−4^) and the lyso-phosphocholine PC_a_20:4 (p = 1.4×10^−3^) display marked negative associations with diabetes, followed by many other PC-species with poly-unsaturated fatty acids (PUFAs) in their lipid side chains, although with lower, but still significant strengths of association (PC_ae_C40:1, PC_ae_C36:3, PC_ae_C38:5, …; see Table S2). On the other hand, phosphatidylethanolamines with similar lipid side chain compositions (PE_aa_C34:2, PE_aa_C36:2, PE_aa_C38:4, …) display an increase in the diabetes group. For example, statistically significant associations (p = 2.6−8.3×10^−5^) are observed in particular when ratios with beta-hydroxyisovalerate are considered. Concentrations of medium-chain length fatty acids and arachidonate are on average lower in the diabetes group, while other long chain fatty acids are higher, including a number of PUFAs, such as the essential fatty acids linolate and linolenate (Table 4). Note in particular that arachidonate is incorporated into the lipid side chains of the above mentioned (lyso)phosphatidylcholines PC_aa_C34:4 and PC_a_20:4.

**Table 4:** Medium chain-length fatty acids and arachidonate are on average lower in the diabetes group, while long chain fatty acids are higher; shown are all fatty acids that associate with diabetes in at least one ratio pair combination with a q-value <5% and a p-gain >1.

Lower in diabetes	Higher in diabetes
caproate (6:0)	myristate (14:0)
heptanoate (7:0)	palmitate (16:0)
pelargonate (9:0)	2-hydroxypalmitate
10-undecenoate (11:1n1)	margarate (17:0)
arachidonate (20:4n6)	10-heptadecenoate (17:1n7)
	stearate (18:0)
	2-hydroxystearate
	oleate (18:1n9)
	linoleate (18:2n6)
	linoleamide (18:2n6)
	linolenate (18:3n3 or 6)
	eicosenoate (20:1n9 or 11)
	dihomo-alpha-linolenate (20:3n3)
	adrenate (22:4n6)

The branched chain amino acids (BCAAs) leucine, isoleucine, and valine as well as their gamma-glutamyl-derivates are all increased in the diabetes group (Table 5). We observe highly increased concentrations of the ketone body 3-hydroxybutyrate (BHBA) in the diabetes group (+53.9% when measured on a MS platform, +40.6% on the NMR platform). At the same time, the acetate concentrations are lower, as indicated by the ratio between acetate and BHBA (p = 1.1×10^−4^). Association with acetate alone was not significant. 3-indoxyl sulfate (IS) concentrations are on average 42.8% higher in the diabetes group (p = 1.7×10^−4^). Additionally, creatinine concentrations are found to be increased by over 16% in the diabetes group. The association of the cysteine to creatinine ratio with diabetes (p = 1.1×10^−6^) increases by a factor of 289.5 when compared to the association of the single metabolites alone, with cysteine concentrations found decreased in the diabetes group and creatinine values increased. Similarly, the association of ratios between cysteine and IS (p = 6.8×10^−7^) increases by a factor of 242.7.

**Table 5:** Difference of average branched chain amino acids (BCAA) concentrations between the diabetes and the control group; a positive value %-change indicates higher concentrations in the diabetes group; where metabolites that were measured on more than one platform, results from these platforms are presented separately.

Metabolite	%-change	p-value
glutamylvaline [M]	26.4%	2.9×10^−4^
gamma-glutamylisoleucine [M]	27.8%	6.7×10^−4^
isoleucine [C]	13.6%	7.8×10^−3^
(iso)leucine [B]	8.3%	0.017
(iso)leucine [C]	13.5%	0.017
leucine [C]	13.5%	0.039
gamma-glutamylleucine [M]	12.3%	0.055
valine [B]	7.0%	0.059
leucine [M]	7.2%	0.087
isoleucine [M]	7.9%	0.095
valine [C]	7.4%	0.15
valine [M]	2.5%	0.55

Some metabolites were detected in only a limited number of study participants. Salicyluric glucuronide was detected in 16 of 40 subjects with diabetes and in 8 of 60 controls (two-sided exact Fisher test: p = 0.0037). In three patients with diabetes, both pioglitazone and hydroxypioglitazone were detected, confirming the intake of diabetes-specific medication; whereas none were observed in the control group. Erythritol, a naturally-derived sugar substitute, was found to be associated with diabetes in ratios with cysteine. Phenylacetate was detected in 29 of 40 participants with diabetes and only in 31 of 60 controls (p = 0.041). Finally, kynurenine levels are found to be 14.6%–21.8% higher in the diabetes group. The bile acid cholate was detected in only 28 of 40 subjects with diabetes and in 53 of 60 controls (two-sided p = 0.036). Deoxycholate was found in 27 of 40 persons with diabetes and only in 27 of 60 controls (p = 0.040). Gamma-muricholate (hyocholic acid) was detected in only 4 of 40 patients with diabetes and in 16 of 60 controls (p = 0.045).

Small peptides can be detected by the present technology when present in sufficient concentrations. Fibrinogen alpha chain peptides (ADpSGEGDFXAEGGGVR and ADSGEGDFXAEGGGVR) were found to associate with diabetes (p-value<0.05), albeit at levels that were not significant after correcting for multiple testing, and thus require future replication. However, mentioning this marginal association here is noteworthy since fibrinogen is a blood-borne glycoprotein. Various cleavage products of fibrinogen regulate cell adhesion and spreading, display vasoconstrictor and chemotactic activities, and are mitogens for several cell types. There is evidence of increases in fibrinogen peptide in the context of atherosclerotic plaque progression [36], [37].

## Discussion

Before discussing the results, we should note the caveats of the present study. These are mainly due to the history of how this study was conducted. Consequently, we consider this work as a pilot for future studies under more controlled conditions. Although the concept of metabolomics as the science of measuring ideally all small metabolites in a body fluid has been pioneered as early as 1971 by Linus Pauling [38], performing metabolomics in a high-throughput setting is a relatively young science. One of our objectives was therefore to evaluate its potential for use in larger population studies [39]. Therefore, we submitted blood samples that had been collected previously to different commercial metabolomics providers in order to evaluate the signals that can be detected when the data is compared to the different KORA phenotypes [29], [30]. Unfortunately, standard examinations and collection of blood samples for metabolomics were separated by about one to two years (see methods), introducing some uncertainty with respect to parameters that were measured at baseline only (e.g. body mass index, HbA1c, waist-hip-ratio, questionnaires) and not at the time blood was sampled for metabolomics. Some of these parameters, for instance “current medication”, are analyzed as covariates and results are provided as Table S4, but should be interpreted considering this caveat. However, blood lipid parameters were measured in the same samples as were the metabolites.

Some of the metabolites measured on more than one platform show only moderate levels of agreement (see results). It is not our objective here to identify the exact reasons behind these differences, as this would require access to proprietary information of commercially operating companies. However, some hypothesis on possible explanations for the less concordant measurements is worth noting. Due to material availability serum samples had to be used for MS, while only sufficient plasma samples were available for NMR measurements; also the available sample volume was suboptimal for NMR; the two MS platforms are not using identical fractionation pattern for the identification of their metabolites, nor do they use identical sample preparation methods, so that dependent on the platform, in some cases ion suppression or interference with signals from other metabolites may occur. Finally, for funding reasons, we had to limit this study to 100 male individuals aged above 55 years. Keeping these caveats in mind, we will now discuss the results obtained in this study (summarized in Table 6).

**Table 6:** Overview of findings.

Observation (relative to the control group)	Interpretation
Increased sugar metabolites	Impaired insulin sensitivity
Reduced 1,5-anhydroglucitol	Short term marker of glycemia
Decreased PCs, increased PEs	Compatible with lower HDL/total cholesterol and higher triglyceride levels in patients with diabetes
Decreased medium chain-length fatty acids and arachidonate, increased longer chain fatty acids and PUFAs	Modification of lipid homeostasis
Increased BCAAs	Increase in glucose-alanine cycle = Impaired short-term metabolic control!
Increased 3-hydroxybutyrate	Marker of ketosis
Increased creatinine, 3-indoxyl sulfate, detection of phenylacetate, strengthened association for cysteine to creatinine and to 3-indoxyl sulfate ratios	Marker of nephritis, kidney function impairment and nephropathy
Detection of pioglitazone and hydroxypioglitazone, salicyluric glucuronide, and erythritol	Detection of diabetes-specific xenobiotics
Reduced detection of cholate and gamma-muricholate, increased detection of deoxycholate	Higher activity of primary into secondary bile acid conversion by gut flora?

Differences in the clinical biochemistry parameters between the diabetes and the control group are relatively modest (Table 1). Cases with diabetes had a higher BMI and waist-hip-ratio than control subjects. However, they did not exhibit strongly adverse lipid profiles. Total cholesterol and LDL were actually lower in the diabetes group, possibly a consequence of lipid lowering medication. The average HbA1c value, albeit measured at the base examination, was 5.95% in the diabetes group versus 5.29% in the control group suggesting that the study participants controlled their disease state relatively well. An important point to note is that the metabolic signals that can be identified in such a population study under non-clinical conditions could be expected to be much more pronounced in a clinical setting and with undiagnosed diabetes patients or poorly controlled diabetics.

### Carbohydrate metabolism

Glucose and many related carbohydrate metabolites were readily confirmed as a diabetes biomarker. Modified hexoses such as N-acetylglucosamine, deoxyglucose and glucuronic acid were all detected at higher serum concentrations in the diabetes group, mirroring the increased supply of glucose as a precursor for their biosynthesis. The same effect is observed by elevated maltose levels. Alterations of glycolipid and glycoprotein biosynthesis and degradation could possibly lead to increased serum concentrations of complex sugars, containing N-acetylglucosamine and sialic acid moieties. 1,5-anhydroglucitol (1,5-AG), the 1-deoxy form of glucose is a short-term glycemic marker [40], [41]. 1,5-AG is currently used to monitor glycemic control in patients with diabetes and is a good positive control of this study [42]. Its renal loss, which is stimulated in hyperglycemic conditions by glycosuria, results in lowered plasma concentrations. In agreement with these observations we found a significant decrease of average 1,5-AG concentrations in participants with diabetes when compared to the control group (Figure 2). A detailed analysis of such a complex set of sugar metabolites may be combined in future studies with additional phenotypic information, such as lifestyle and nutrition pattern, to investigate the metabolism of these sugar metabolites more specifically in the context of their role in diabetic arteriosclerosis and hyperglycemia.

### Branched chain amino acid (BCAA) metabolism and gluconeogenesis

The BCAAs leucine, isoleucine, and valine as well as their gamma-glutamyl-derivates are all increased in the diabetes group, indicating an impaired short-term metabolic control [43], i.e. peripheral tissues such as skeletal muscle may have lost their ability to react to the presence of energy sources by selective uptake mechanisms. These observations are concordant with what is measured in diabetic mice, where increased plasma levels of BCAAs were also found [15], as well as in experimentally diabetic rats [44] and in insulin-dependent type 1 diabetic human patients [45]. Gamma-glutamyl derivates are formed in the glutathione-dependent transport of certain amino acids. This indicates that not only the levels of these amino acids differ between the groups, but that also an effect on the transport of these amino acids may be observed. BCAAs contribute to glucose recycling via the glucose-alanine cycle [46]. There is a continuous flux of BCAAs from visceral tissues through the blood to skeletal muscle where transamination of the BCAAs provides the amino group for production of alanine from pyruvate with a corresponding movement of alanine from muscle to liver to support hepatic gluconeogenesis [47]. Under normal conditions, alanine arising from BCAA nitrogen likely accounts for 25% of gluconeogenesis from amino acids [48]. Diabetic db-/db- mice present clear evidence of increased gluconeogenesis, visible in strongly decreased concentrations of the gluconeogenic amino acids alanine, glycine, and serine [15]. However, such changes are not observed here. Glycine concentrations are not significantly different between the two groups; alanine and serine concentrations are even 6–10% higher in the diabetes group. Obviously, study participants with diabetes do not show significantly increased gluconeogenesis (limited validity of the mouse model) or the consumption of the glucogenic amino acids is better compensated by increased proteolysis.

### Perturbation of lipid metabolism

It is known that the characteristic features of dyslipidemia in humans with type 2 diabetes are high plasma triglyceride concentration, low HDL cholesterol concentrations and increased concentration of small dense LDL-cholesterol particles, while total cholesterol is not increased in patients with diabetes. These lipid changes in these individuals may be due to an increased free fatty acid flux secondary to insulin resistance [49]. However, the perturbations observed here in lipid metabolism reflect the state of already treated diabetes. Association studies with blood lipid parameters [Adamski et al., unpublished data, [50]] show that many PC species associate with HDL and total cholesterol levels while PE species associate with triglyceride levels. In this study, we observed lower phosphatidylcholine (PC) and higher phosphatidylethanolamine (PE) concentration in the diabetes group matches the lower HDL and total cholesterol levels and higher triglyceride levels in this group, indicating that these glycerophospholipids may provide a more differentiated view of the shifted lipid homeostasis in patients with diabetes as what can be obtained from the bulk blood cholesterol and triglyceride parameters alone. Consistent with this finding, Gall et al. [34] observed reduced levels of multiple acylglycerophosphocholine species that were highly correlated with insulin resistance as measured by the euglycemic clamp.

### Mild signals of ketosis can be discerned

The two common ketones produced in humans are acetoacetic acid and β-hydroxybutyrate [51]. Acetoacetic acid was not measured in this study. In the diabetes group we observe highly increased concentrations of the ketone 3-hydroxybutyrate (BHBA). At the same time, the acetate concentrations are lower, as indicated by the ratio between acetate and BHBA. Traditionally, in clinical practice hyperketonemia and diabetic ketoacidosis were predominant medical conditions in persons with type 1 diabetes. However, newer data reveal that hyperketonemia or diabetic ketoacidosis can also co-exist with hyperglycemia in patients with type 2 diabetes [52]. Ketogenesis, the formation of ketone bodies from lipid breakdown and deamination of amino acids, occurs after depletion of the hepatic glycogen pool. In the case of diabetes, this can be related to glucose defective absorption, related to insulin deficiency. The concentrations of ketones observed here probably do not correspond to a state of diabetic ketoacidosis, but indicate that the diabetes group shows signals of mild ketosis. A similar phenotype has previously been observed in a clinical biomarker study on metabolic syndrome patients (three or more ATP criteria) where the carnitine ester of BHBA was found at significantly elevated concentrations as compared to healthy controls (Weinberger, unpublished data).

### Early signals of impaired renal function

3-indoxyl sulfate (IS), which is higher in the diabetes group, is metabolized by the liver from indole, which is produced from tryptophan by the intestinal flora. IS shows nephrotoxicity and is a stimulating factor for progression of chronic renal failure (CRF) [53], [54]. Additional evidence for the onset of diabetic nephropathy are creatinine concentrations, which are found to be increased >16% in the diabetes group. Creatinine is produced in muscle and is often increased in diabetic nephropathy. Assuming that higher creatinine and IS values are indicators for impaired renal function, these findings are in agreement with previous work that shows that glomerular filtration rate (GFR) is the rate-limiting factor for renal clearance of cysteine in patients with diabetes but without overt nephropathy, and that hyperfiltration explains lower than normal mean plasma total cysteine concentrations in patients with diabetes [55]. Moreover, another study [56] suggests that an observed increase of plasma homocysteine concentration with age could be partly due to the deterioration of renal function. Our results suggest that the cysteine to creatinine and the cysteine to IS ratios are indicators of deterioration of renal function in diabetes patients and support the idea that endogenous amino acid catabolites, such as IS, may play a significant role in the progression of chronic renal failure. Furthermore, phenylacetate was more often detected in the diabetes group in this study. Phenylacetate is a carboxylic acid ester that has been found in the biofluids of patients with nephritis [57]. Taken together, these results suggest that signals of the onset of renal failure can be detected in the diabetes group, although, as we have already pointed out in the case of ketosis, the concentrations we observe here are still in the range that would not yet be considered as pathogenic, but rather represent “mild” or “early” phenotypes. These findings now need to be further verified by a quantitative analysis from retrospective studies, especially in the light of recent findings that L-cystine stone prevention by rational design of crystal growth inhibitors may be possible [58].

### Diabetes-specific medication can be identified

In our study salicyluric glucuronide was more frequently detected in subjects with diabetes. Salicyluric glucuronide is the product of the hydrolysis of acetylsalicylic acid in liver, blood and some other organs. Detection of increased levels of this drug metabolite is plausible considering subjects with diabetes more frequently use drugs such as acetylsalicylic acid due to their high risk for cardiovascular complications [59]. Furthermore, both pioglitazone and hydroxypioglitazone were detected in individuals with diabetes only, confirming the intake of diabetes-specific medication. In addition, the association with erythritol could indicate a more frequent use of sweetener by patients with diabetes. Thus, metabolomics does not only allow the detection of naturally occurring diabetes-related compounds, but also allows for an external assessment of medication and intake of other xenobiotic substances when such information is not available otherwise.

### Bile acids (BAs) that can be transformed by gut bacteria are less often detected in patients with diabetes

Cholate was detected more frequently in the control group than in the diabetes group, while the opposite was true for deoxycholate. Along with chenodeoxycholic acid, cholic acid is one of two primary bile acids produced exclusively in the liver where it is synthesized from cholesterol. Deoxycholate, also known as cholanoic acid (3α,12α-dihydroxy-5β-cholanate), is one of the secondary bile acids and constitutes a significant part of the circulating BA pool in humans (35%). These secondary BAs can be either passively absorbed to enter the BA pool or being excreted in the faeces. The fecal loss of BAs, which is compensated for by de novo BA biosynthesis in the liver to maintain pool size, represents a major route for cholesterol turnover. Therefore, the present findings may indicate that patients with diabetes exhibit alterations in the composition of the bile acid pool, and their related biosynthetic pathways, possibly including a higher rate of conversion of primary to secondary bile acids by the gut microflora. Stemming from the data observed here, this hypothesis is in agreement with the fact that the gut microbiota composition can be different between controls and individuals with diabetes [60].

### Limitations of the present study

The challenges of interpreting metabolic profiling data in a human population include the following two points. First, a human population is expected to display a much more heterogeneous metabolic makeup as compared to inbred animal models under laboratory conditions, with different environmental factors, lifestyle and genetic background being the major contributors. Second, human patients with diabetes will react to their diagnosed diseased state in many ways, for example by differentially changing their nutritional habits, level of physical exercise, and by intake of a variety of anti-diabetic and cardiovascular-protective medications. Despite these pronounced heterogeneities within a human diabetic population, the present study with a limited sample size was nevertheless able to detect many differences between patients and controls in metabolic profiles from different pathways, lending deeper insights and increased sensitivity for detection of a diabetic metabolic state. Many observations presented here are consistent with what is known about diabetes – but not necessarily at clinical levels. The participants of this study represent a random sample of the general population, not a specific selection of diabetes patients with acute or clinical symptoms. They are more likely to have their diabetes under control, thereby representing relatively mild, or possibly earlier phenotypes.

### Conclusion

**Figure 3:**
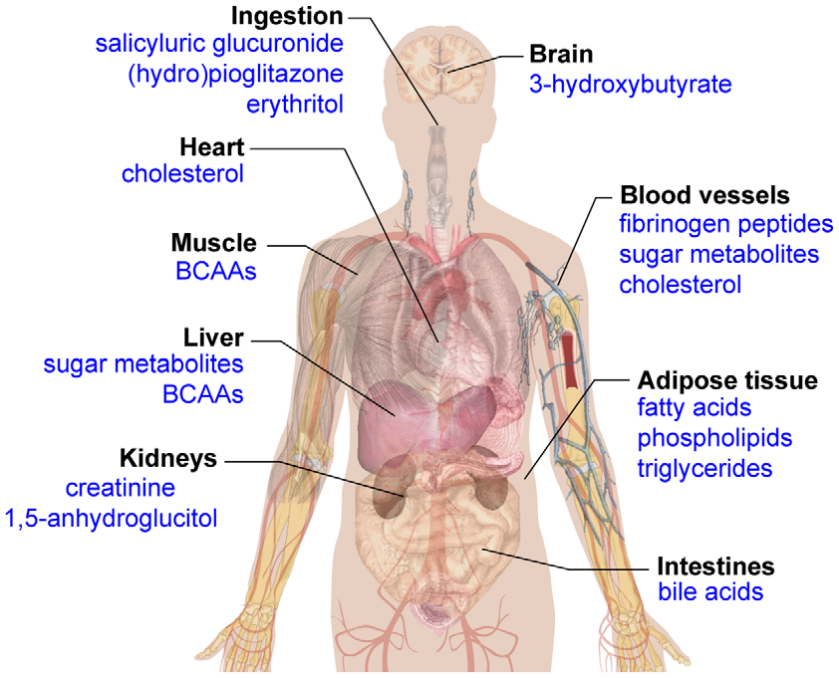
A systemic view of metabolic markers that associate with diabetes in this study. The coverage of the metabolome's diversity allows the detection of systemic metabolic imbalances, thereby providing a disease specific picture of human physiology.

Our study represents the first multi-platform approach to the metabolome-wide analyses of diabetes in a general population. The identification of biomarkers allowing prediction of disease progression and its complications from such studies would be certainly beneficial. However, for the caveats discussed above, we feel that this study should be considered as a pilot for future work. One major finding of our work is the identification of a series of known, and also some novel, deregulated metabolites that associate with diabetes under sub-clinical conditions in the general population. These metabolites have been discovered by integrative metabolomics applying different platforms including nuclear magnetic resonance (NMR) and mass spectrometry (MS). Out of the multitude of metabolites measured, a holistic view of differences reflecting global variations in pathophysiology emerges from our study. The coverage of the metabolome's diversity allows the detection of systemic metabolic imbalances, thereby providing a disease-specific picture of human physiology (Figure 3). A pronounced increase in the sample size in future studies will likely allow for further detection of other metabolites of unrecognized associations with diabetic pathways. Finally, our study shows how functional metabolomics can contribute to obtaining a more sophisticated classification of the disease as well as rational optimization of diagnostic and treatment options, as recently suggested by Bain et al. [4].

## Supporting Information

Text S1 Description of the metabolomics companies' QC processes.
(0.16 MB PDF)

Table S1 Correlation data (R) for metabolites that were measured on more than one platform.
(0.03 MB XLS)

Table S2 Association data for all 482 metabolite concentrations with diabetes state.
(0.21 MB XLS)

Table S3 Association data from metabolite concentration ratios with diabetes state.
(0.86 MB XLS)

Table S4 Influence of covariates on the association with all 482 metabolite concentrations.
(0.58 MB XLS)

Figure S1 Distribution of the Pearson correlation coefficient (R2) between the different platforms. The full dataset with all available cross-platform correlation coefficients is provided in Table S1.
(0.08 MB PDF)

Figure S2 Selected examples of metabolites that were measured on multiple platforms; top row: alanine concentrations measured on Biocrates (FIA-MS), Chenomx (NMR), and Metabolon (GC-MS) platforms; middle row: proline concentrations measured on Biocrates (FIA-MS), Chenomx (NMR) and Metabolon (LC-MS) platforms; bottom row: 3-hydroxybutyrate measured on Chenomx (NMR) and Metabolon (GC-MS) platforms, acetylcarnitine measured on Biocrates (FIA-MS) and Metabolon (LC-MS) platforms, and arachidonic acid measured on Biocrates (LC-MS) and Metabolon (LC-MS) platforms. Units of Biocrates and Chenomx are in µM (absolute quantification), Metabolon reports ion counts (relative quantification). The full dataset with all available cross-platform correlation coefficients is provided in Table S1.
(0.15 MB PDF)
