# Supplementary material for: Metabolic Footprint of Diabetes: A Multiplatform Metabolomics Study in an Epidemiological Setting
Source: PLoS One. 2010 Nov 11;5(11):e13953. doi: 10.1371/journal.pone.0013953 (PMC2978704; doi:10.1371/journal.pone.0013953)
Supplement: Text S1 — Description of the metabolomics companies' QC processes. (0.16 MB PDF) [file pone.0013953.s001.pdf]

SUPPLEMENTAL TEXT

"Metabolic footprint of diabetes:

A multiplatform metabolomics study in an epidemiological setting"

Karsten Suhre *et al*.

Description of the metabolomics companies' QC processes

Biocrates QC: The method applied by Biocrates is proven to be in conformance with 21CFR (Code of Federal Regulations) Part 11, which implies proof of reproducibility within a given error range. In addition to the company's internal QC tests, we have conducted independent replication experiment on a subset of the metabolite panel that was used in this study. The majority of metabolites had a CV lower than 10%, higher values of CV are generally observed for metabolites that are closer to the detection limit and at very low concentrations (see Figure below).

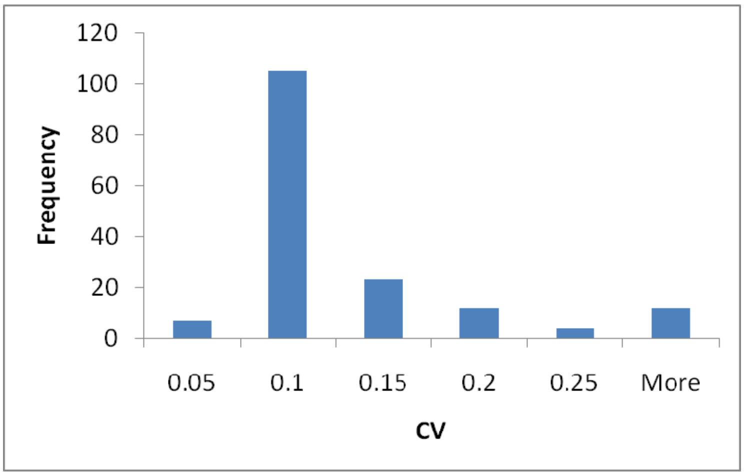

Experimental variance (CV) determined based on a study with 45 technical replicates of identical human serum measured on 9 different Biocrates AbsoluteIDQ kit plates (5 replicate samples/kit).

Chenomx QC: Targeted Profiling of the samples was done in a blinded manner by three analyst using Chenomx NMR Suite. A full peer review was performed by a different analyst for each of the NMR spectra that was annotated. Finally a third and final overall review was done to account for any gross errors such as missing values and mixed identifications. The reproducibility of the data can be broken down into two sources of error. Error or variability introduced by the NMR machine itself, and the variability introduced by Targeted Profiling. The error from the NMR machine is negligible. The noise percent for these spectra measured on a 400 MHz spectrometer for 1D Proton NMR using 32 scan was calculated to be 0.5%. The coefficient of variation of the error introduced by using Chenomx

NMR Suite to calculate absolute concentrations of metabolites is on average less than 5% for a trained user for each metabolite, as reported by Chenomx internal testing. The error varies for different metabolites and different types of mixtures. The error of identifying metabolites using the Chenomx library is more difficult to measure since there isn't a practical way to test this using standard mixture. The results reported for this project only contained positively identified metabolites.

Metabolon QC: Instrument variability was determined by calculating the median relative standard deviation (RSD) for the internal standards that were added to each sample prior to injection into the mass spectrometers. Overall process variability was determined by calculating the median RSD for all endogenous metabolites (i.e., noninstrument standards) present in 100% of the Matrix technical replicate samples. For this purpose, a small aliquot of each experimental sample for a specific matrix is obtained from each experimental sample and pooled together as a “Client matrix” (CMTRX) to serve as technical replicates (see Figure below). Aliquots of these CMTRX samples are injected throughout the platform day run and serve as technical replicates. As such, the variability in the quantitation of all the consistently detected biochemicals in the experimental samples can be monitored. With this monitoring, a metric for overall process variability can be assigned for the platform’s performance based on the quantitation of metabolites in the actual experimental samples. Values for instrument and process variability meet Metabolon’s acceptance criteria as shown in the table below.

QC Sample	*Measurement*	*Median RSD*
Internal Standards	Instrument Variability	5%
Endogenous Biochemicals	Total Process Variability	10%

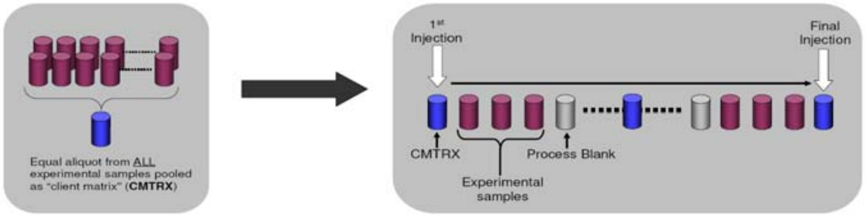

Preparation of client specific technical replicates (CMTRX). A small aliquot of each client samples is pooled (blue cylinder). This pooled sample is then injected periodically throughout the series of injections that comprise the experimental and other QC samples during a platform day run. The quantitation of the panel of biochemicals detected in these injections can then be compared to produce an estimate of process variability.
