# Supplementary figures and images for: Metabolic Footprint of Diabetes: A Multiplatform Metabolomics Study in an Epidemiological Setting
Source: PLoS One. 2010 Nov 11;5(11):e13953. doi: 10.1371/journal.pone.0013953 (PMC2978704; doi:10.1371/journal.pone.0013953)

# Distribution of R<sup>2</sup> values

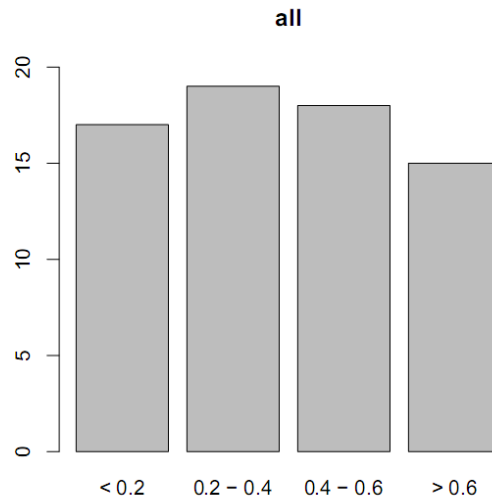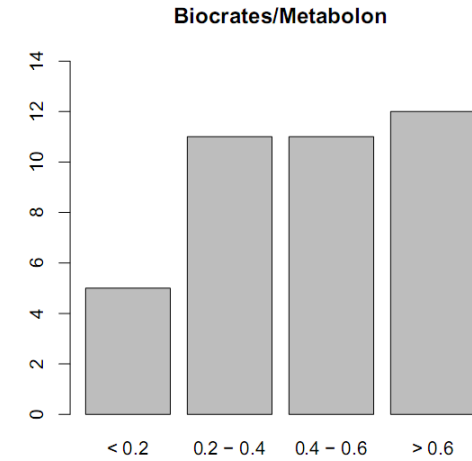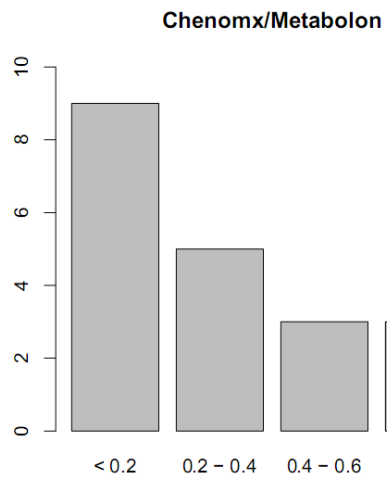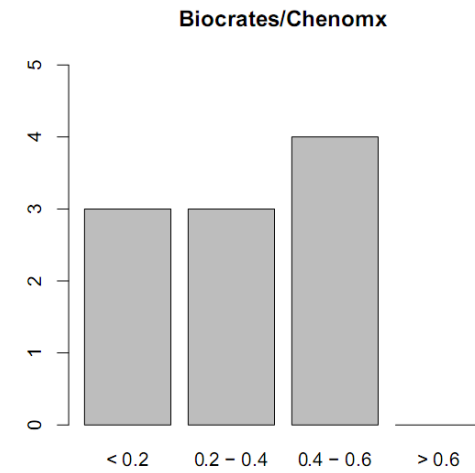

Supplement: Figure S1 — Distribution of the Pearson correlation coefficient (R2) between the different platforms. The full dataset with all available cross-platform correlation coefficients is provided in Table S1. (0.08 MB PDF) [file pone.0013953.s006.pdf]

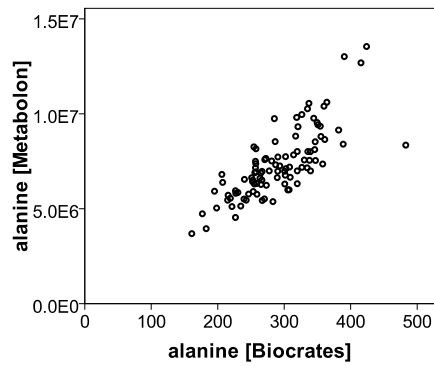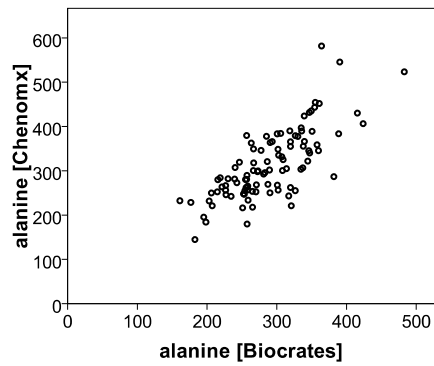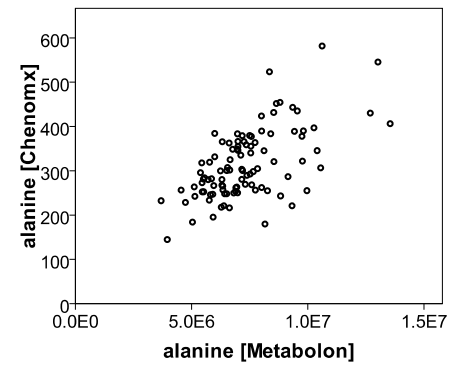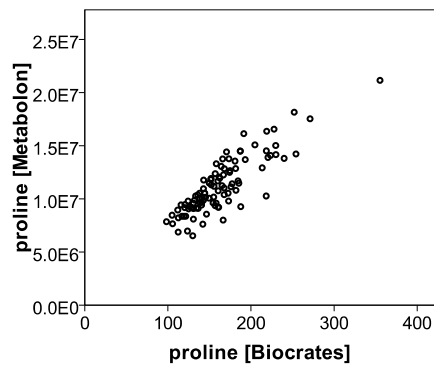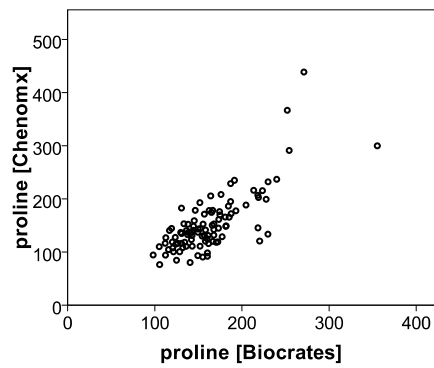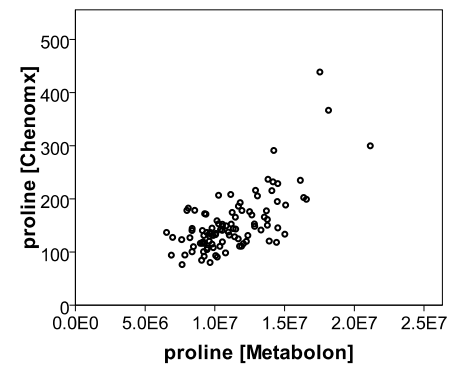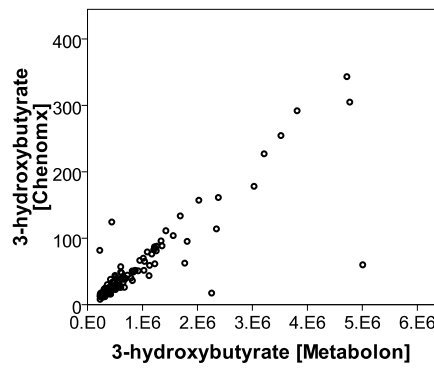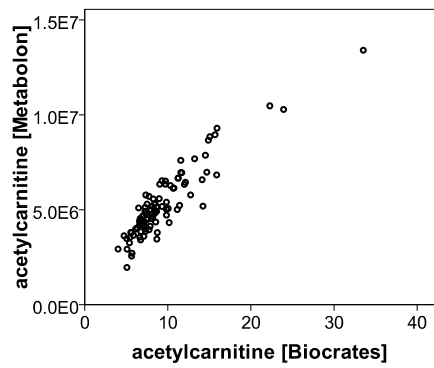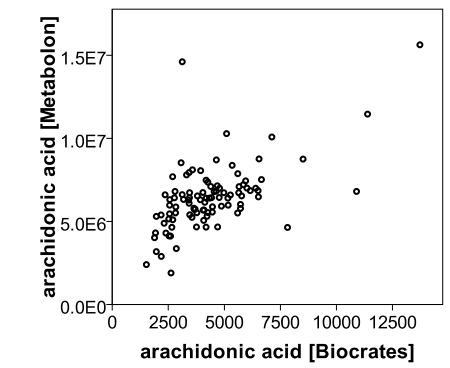

Supplement: Figure S2 — Selected examples of metabolites that were measured on multiple platforms; top row: alanine concentrations measured on Biocrates (FIA-MS), Chenomx (NMR), and Metabolon (GC-MS) platforms; middle row: proline concentrations measured on Biocrates (FIA-MS), Chenomx (NMR) and Metabolon (LC-MS) platforms; bottom row: 3-hydroxybutyrate measured on Chenomx (NMR) and Metabolon (GC-MS) platforms, acetylcarnitine measured on Biocrates (FIA-MS) and Metabolon (LC-MS) platforms, and arachidonic acid measured on Biocrates (LC-MS) and Metabolon (LC-MS) platforms. Units of Biocrates and Chenomx are in µM (absolute quantification), Metabolon reports ion counts (relative quantification). The full dataset with all available cross-platform correlation coefficients is provided in Table S1. (0.15 MB PDF) [file pone.0013953.s007.pdf]
